# From BJBMS to Biomolecules and Biomedicine: A new chapter

**DOI:** 10.17305/bb.2025.12867

**Published:** 2025-06-27

**Authors:** Faruk Skenderi, Lamija Mlaco, Muzafer Mujic, Semir Vranic

**Affiliations:** 1 Biomolecules and Biomedicine, Sarajevo, Bosnia and Herzegovina; 2 Association of Basic Medical Sciences of Federation of Bosnia and Herzegovina, Sarajevo, Bosnia and Herzegovina; 3 College of Medicine, QU Health, Qatar University, Doha, Qatar

The scientific community is continually evolving, driven by advancements, shifting priorities, and growing demands for global dissemination of knowledge. A clear example of successfully adapting to these demands is the transition from the Bosnian Journal of Basic Medical Sciences (BJBMS) to Biomolecules and Biomedicine (BB) in 2023 [1]. This strategic move symbolizes a significant step forward, expanding the journal’s global reach and scientific scope.

## Understanding journal impact factors (JIF)

Various journal metrics, such as the Journal Impact Factor (JIF) and CiteScore, continue to be pivotal measures for assessing the influence of scholarly journals. Calculated based on recent citations to a journal’s published articles, JIF and CiteScore provide a quick indication of the relative importance of journals within their respective fields. However, it’s essential to remember that JIF and CiteScore alone do not fully capture a journal’s quality or influence.

## Transition in impact factors and CiteScore: A closer look

**Figure 1.**
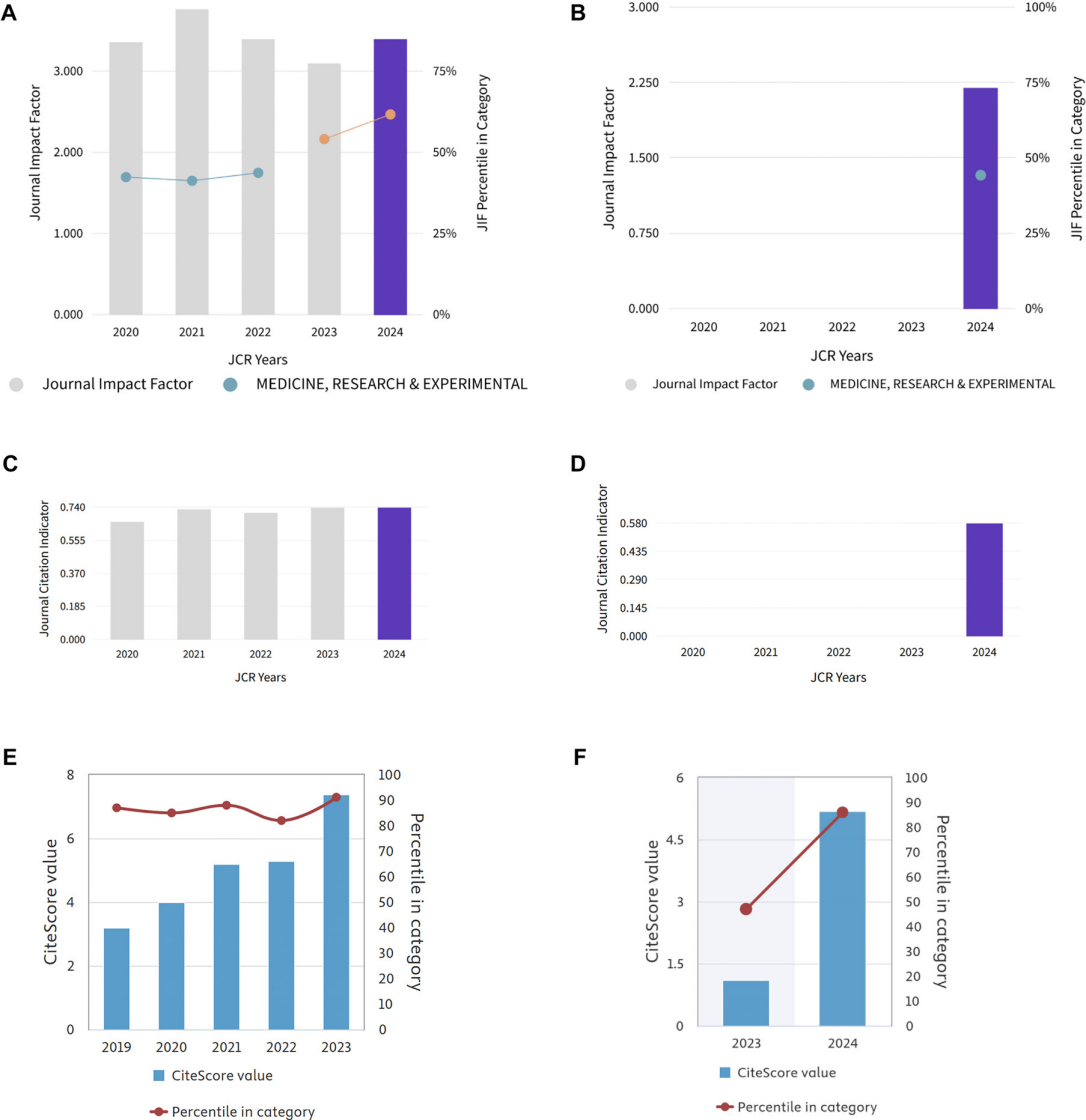
**Performance metrics before and after rebranding from Bosnian Journal of Basic Medical Sciences (BJBMS) to Biomolecules and Biomedicine (BB).** (A) The five-year Journal Impact Factor (JIF) trend for BJBMS from 2020 to 2023 is represented by grey bars, with the 2024 JIF recalculated by Clarivate under the unified title indicated by the purple bar. The orange line illustrates the journal's percentile rank within the Medicine, Research & Experimental (MED-RE) category (right-hand *y*-axis). (B) The first JIF for BB (2024; purple bar) and its MED-RE percentile (teal marker) are presented. Earlier years are omitted as no JIF was calculated for BB prior to the 2023 publication year. (C) The Journal Citation Indicator (JCI) progression for BJBMS from 2020 to 2023 is depicted in grey, with the corresponding 2024 value shown in purple. (D) The initial JCI for BB in 2024 is marked in purple; previous years are not applicable. (E) The evolution of CiteScore for BJBMS from 2019 to 2023 is illustrated, with blue columns representing annual CiteScores and the maroon line indicating the percentile within the Scopus General Medicine category. (F) CiteScore for BB during its first two tracked years (2023--2024) is shown, with blue bars representing CiteScore values and the maroon line tracing the percentile within the category. Grey bars indicate historical values under the former journal title, while purple bars represent the first metrics published after rebranding. Percentile lines and markers utilize the secondary (right-hand) *y*-axis in each panel. All data are sourced from Clarivate Journal Citation Reports^TM^ 2024 (panels A--D) and Elsevier Scopus 2024 (panels E and F).

Calculating JIF for 2024 for BJBMS and BB is a bit more complicated this year. Namely, for JIF 2024, only citations to articles from 2022 and 2023 count towards the JIF, and this implies overlap between the old title and new title articles. Under the old title (BJBMS), articles published in 2022 achieved a commendable JIF of 3.4 (Q2) in 2024 (Figure 1A), reflecting a well-established citation base (Figure 1C). Following the change to Biomolecules and Biomedicine, articles published in 2023 achieved a JIF of 2.2 (Q3), a slight decline reflecting the initial phase post-transition (Figures 1B and 1D). Data from Clarivate’s Journal Citation Reports (2024) confirms that such transitional phases often result in temporary metric adjustments.

In 2024, Biomolecules and Biomedicine achieved a CiteScore: 5.2, SJR: 0.872, and SNIP: 0.938, which places it #91/668 (Q1) in the General Medicine category, and #69/225 (Q2) in the Biochemistry, Genetics and Molecular Biology category (Figures 1E and 1F).

Newly established journals or those undergoing major rebranding typically present lower initial JIFs. For instance, BJBMS maintained a higher JIF (3.4) compared to BB’s initial JIF (2.2), reflecting shorter citation windows post-rebranding. Indeed, early-stage impact factors often underestimate the long-term citation potential and influence of newly branded journals. Metrics like Eigenfactor (BJBMS: 0.00171 vs. BB: 0.00058) and Article Influence Score (BJBMS: 0.734 vs. BB: 0.482) are expected to gradually rise as citation networks and indexing visibility solidify.

## The benefit of viewing combined impact factors

Although official Journal Citation Reports do not combine impact factors across title changes, considering both BJBMS’s strong historical metrics and BB’s initial impact provides a clearer picture of sustained quality and potential growth. Notably, the current impact factors for both BJBMS (3.4) and BB (2.2) each represent citations to articles published within only one year, contrasting with the traditional two-year citation window of regular JIF calculations. A combined approach, averaging these two values, yields a combined impact factor of approximately 2.8. Additionally, considering BJBMS’s ranking in the second quartile (Q2, 61.8 percentile) and BB’s third quartile (Q3, 44.4 percentile) provides a broader context, reflecting sustained influence and competitive standing within the field despite the transition. This combined perspective highlights continuous strength and promising potential for future growth.

## Commitment to open access

A hallmark of both BJBMS and now BB is their robust commitment to Open Access (OA). According to the latest data, BJBMS published 98.97% of its content as open access, while BB continues strongly at 94.14% OA content. The shift towards open access significantly enhances global visibility and accessibility of cutting-edge biomedical knowledge.

## Expanding global influence and collaboration

BB demonstrates remarkable international growth. Contributions now span globally prominent institutions, including Nanjing Medical University (19 papers), Mayo Clinic (17 papers), and Qatar University (18 papers), broadening the journal’s scope and international relevance. This global collaboration enriches content quality and expands readership, setting the stage for future growth in scientific influence.

## Increasing frequency, expanding visibility

Another strategic advancement is the increase in publication frequency from four issues per year (BJBMS) to 12 issues annually under BB. This shift significantly boosts the journal’s visibility, accelerates the dissemination of impactful research, and enhances its overall potential for citations and influence.

## Looking beyond metrics: quality matters

While metrics remain essential, the qualitative aspects of journal management—rigorous peer review, innovative content, transparency, and ethical standards—continue to underpin a journal’s reputation. Both BJBMS historically and now BB demonstrate consistent editorial rigor, ensuring published research maintains high standards of scientific integrity.

## Future outlook: Bright prospects and clear advantages

Looking ahead, BB is strategically positioned for substantial growth in citations, global visibility, and scientific impact. The initial transition phase, reflected in slightly lower metrics, is a temporary and common experience. BB’s commitment to rigorous standards, OA dissemination, and expanding international collaboration strongly indicates a bright and promising future.

BB offers numerous advantages and significant achievements, making it an attractive venue for authors. The journal’s strong editorial standards ensure high-quality research through rigorous and transparent peer-review processes. Enhanced global outreach has attracted contributions from prestigious international institutions, amplifying visibility and citation potential for authors. Furthermore, the OA policy guarantees broad dissemination of articles, offering authors greater exposure and impact within the global scientific community. Additionally, an increased publication frequency (12 issues per year) ensures quicker turnaround times from submission to publication, accelerating scholarly communication. Recent positive developments also include the expansion of our editorial board, the establishment of dedicated integrity teams, and the adoption of advanced software tools to enhance manuscript screening and uphold the highest ethical standards.

The transition from BJBMS to BB marks an exciting chapter in biomedical publishing, emphasizing adaptation, global outreach, and strategic growth. Researchers, authors, and readers alike stand to benefit greatly from engaging with this journal as it continues its upward trajectory. BB is not merely continuing the legacy of BJBMS; it is enhancing it, positioning itself prominently within the dynamic landscape of biomedical science publishing.
